# Adsorptive Removal of Cd, Cu, Ni and Mn from Environmental Samples Using Fe_3_O_4_-Zro_2_@APS Nanocomposite: Kinetic and Equilibrium Isotherm Studies

**DOI:** 10.3390/molecules26113209

**Published:** 2021-05-27

**Authors:** Aphiwe Siyasanga Gugushe, Anele Mpupa, Tshimangadzo Saddam Munonde, Luthando Nyaba, Philiswa Nosizo Nomngongo

**Affiliations:** 1 Department of Chemical Sciences, University of Johannesburg, Doornfontein Campus, P.O. Box 17011, Johannesburg 2028, South Africa; gugushe75@gmail.com (A.S.G.); 216051138@student.uj.ac.za (A.M.); munondechimangadzo@gmail.com (T.S.M.); lnyaba@uj.ac.za (L.N.); 2 Department of Science and Innovation (DSI)/National Research Foundation (NRF) South African Research Chair Initiative (SARChI), Nanotechnology for Water, University of Johannesburg, Doornfontein 2028, South Africa; 3 Department of Science and Innovation (DSI)/Mintek Nanotechnology Innovation Centre, University of Johannesburg, Doornfontein 2028, South Africa

**Keywords:** heavy metals, adsorption, kinetics, isotherms, Fe_3_O_4_-ZrO_2_@APS nanocomposite, wastewater

## Abstract

In this study, Fe_3_O_4_-ZrO_2_ functionalized with 3-aminopropyltriethoxysilane (Fe_3_O_4_-ZrO_2_@APS) nanocomposite was investigated as a nanoadsorbent for the removal of Cd(II), Cu(II), Mn (II) and Ni(II) ions from aqueous solution and real samples in batch mode systems. The prepared magnetic nanomaterials were characterized using X-ray powder diffraction (XRD), scanning electron microscopy/energy dispersion x-ray (SEM/EDX) Fourier transform infrared spectroscopy (FTIR) and transmission electron microscopy (TEM). Factors (such as adsorbent dose and sample pH) affecting the adsorption behavior of the removal process were studied using the response surface methodology. Under optimized condition, equilibrium data obtained were fitted into the Langmuir and Freundlich isotherms and the data fitted well with Langmuir isotherms. Langmuir adsorption capacities (mg/g) were found to be 113, 111, 128, and 123 mg/g for Cd, Cu, Ni and Mn, respectively. In addition, the adsorption kinetics was analyzed using five kinetic models, pseudo-first order, pseudo-second order, intraparticle diffusion and Boyd models. The adsorbent was successfully applied for removal of Cd(II), Cu(II), Mn (II) and Ni(II) ions in wastewater samples.

## 1. Introduction

Heavy metal accumulation in the environment is a major threat to public health and to the environment [1]. More commonly, heavy metals come from a variety of sources such as industrial wastes from coal, paper, textile, metal plating, mining operations, tanneries, smelting, alloy and batteries storage industries, among others [2,3]. The most frequently found toxic heavy metals in industrial wastewater include cadmium, chromium; zinc, chromium, copper and lead, among others [2]. Some of these heavy metals, namely, nickel, copper and manganese, are essential to living organisms, however, they are toxic when ingested in excess beyond maximum regulated levels [4]. On the other hand, heavy metals such as lead, and cadmium are known to be extremely toxic and hazardous to human health, even at trace levels [4,5]. Subsequently, these metals are known to have detrimental effects on metabolic processes of human beings and have also been classified as carcinogenic agents [4]. Unlike most organic pollutants that are susceptible to biological degradation, heavy metal ions are not degradable [3]. Therefore, it is necessary for heavy metal concentrations to be monitored and regulated within the environment.

In view of the above, various treatment processes for removal of heavy metals from polluted water have been reported in the literature. These methods include precipitation [6]; membrane filtration [7]; ion exchange [8]; adsorption [9] and co-precipitation [10]. Amongst these methods, adsorption is preferred due to its attractive advantages such as low waste production, easiness, effectiveness and flexibility in design [9]. Furthermore, the use of various sorbents is one of the main attractive features of the adsorption method. Thus, numerous adsorbents have been reported for removal of heavy metals in water. These adsorbents include, ZrO_2_ nanosheets [11]; Chromosorb [12]; fullerene [13]; activated carbon [14,15]; eggshell membrane [16], modified silica gel [17], XAD resins [18] and ion exchange resin [8], among others.

In the adsorption method, the effective removal of heavy metals is predominantly dependent on the properties of the adsorbent surface. For instance, an adsorbent with a large surface area and high porosity is known to have more adsorption sites available for metal ion interaction [19,20]. Conversely, if the surface of the adsorbent is negatively charged at pH’s higher than its point of zero charge (PZC), this becomes beneficial for the adsorption/removal of heavy metals [19]. Recently, due to their unique properties, the use of nanometal oxides as adsorbents for removal of heavy metal has gained more attention in the field of analytical science [21]. The special properties include large surface area, small particles size, resistance to corrosion, non-toxicity and low cost and high chemical stability, among others [9,20,21,22]. Among different kinds of nanometer-sized metal oxides, magnetic nanomaterials are considered potential adsorbents for the adsorptive removal of heavy metals [23]. This is attributed to their size in nano-range, high surface area to volume ratios, super paramagnetism and the unique advantage of easy separation under external magnetic fields [23,24,25,26]. In addition, magnetic nanomaterials have low toxicity, chemical inertness and biocompatibility [27]. In general, any nanomaterial should be stable to avoid aggregation and provide a low deposition rate, to assure their reactivity and mobility [28]. However, this is not the case for magnetic nanoparticles because they tend for form aggregate in solutions [23]. In addition, the disadvantages of pure magnetic nanoparticles include lack of selectivity and unsuitability for complex matrix application [26]. These limitations have been solved by the surface modification using appropriate organic functional groups, leading to the formation of nanocomposites [23,25,29]. This is usually achieved by merging synthetic polymers on inorganic nanoparticles or by adding modified nanoparticles to polymer matrices [29,30]. These modifications have led to high adsorption affinity and enabled specific metal complexation [23,24,25,29,30].

In this study, Fe_3_O_4_-ZrO_2_ nanocomposite functionalized with 3-aminopropyltriethoxysilane was synthesized and used as a nanoadsorbent for the removal of Cd(II), Cu(II), Mn(II) and Ni(II) ions from aqueous solutions. Multivariate studies were performed to determine the factors (such as adsorbent dose and sample pH) affecting the adsorption removal process and obtain their optimum conditions. Then, isotherm and kinetic studies were done and fitted into their relevant models. The adsorbent was then used for removal of Cd(II), Cu(II), Mn (II) and Ni(II) ions from wastewater, acid mine drainage and tap water samples.

## 2. Results and Discussion

### 2.1. Characterization of the Adsorbent

**Figure 1:**
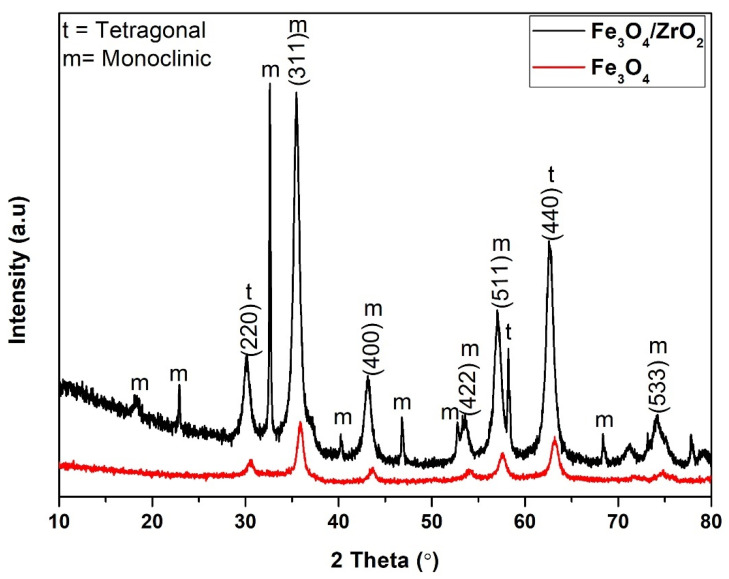
X-ray diffraction patterns for Fe_3_O_4_ nanoparticles and Fe_3_O_4_-ZrO_2_ nanocomposite.

The diffraction patterns of the Fe_3_O_4_ and Fe_3_O_4_-ZrO_2_ nanocomposite are shown in Figure 1. The XRD pattern of crystalline Fe_3_O_4_ (magnetic nanoparticles) MNPs showed characteristics peaks at 30.5°, 35.7°, 43.5°, 54.1°, 57.4°, and 63.2° and 74.9° with corresponding reflection planes of (220), (311), (400), (422), (511), (440) and (533), agree with those reported in literature [23,31]. Subsequently, the same crystal phases were observed on the ZrO_2_-Fe_3_O_4_ XRD pattern with slight shifts in position due to their successful integration with ZrO_2_, forming a ZrO_2_-Fe_3_O_4_ hybrid [32,33]. Interestingly, the crystallinity of the Fe_3_O_4_ MNPs only reduced slightly with the introduction of ZrO_2_, with the hybrid remaining strongly crystalline. The characteristic peaks observed at 18.3°, 23.1°, 32.6°, 35.5°, 40.3°, 46.8°, 53.5°, 57.1°, 68.5° and 74.2° displayed the more dominant monoclinic phase of ZrO_2_ [32,33]. Furthermore, the peaks observed at 30.1°, 58.3° and 62.8° exhibited the tetragonal phase of ZrO_2_, suggesting a mixture of both monoclinic and tetragonal phases [32,33,34]. These deconvolutions affirm the successful integration of the strongly crystalline ZrO_2_-Fe_3_O_4_ nanocomposite which agrees with those reported in the literature [34,35].

**Figure 2:**
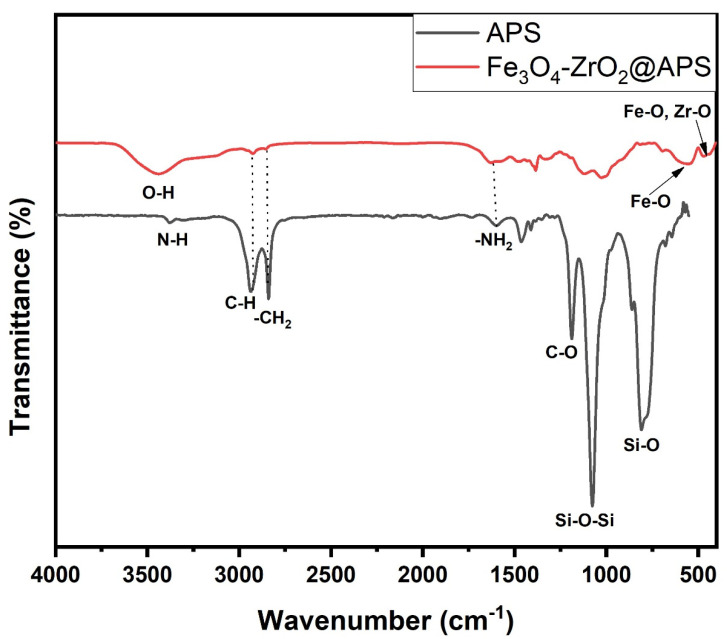
FTIR spectrum of Fe_3_O_4_-ZrO_2_ functionalized with 3-aminopropyltriethoxysilane (Fe_3_O_4_-ZrO_2_@APS).

The FTIR spectrum of the functionalized nanocomposite is displayed in Figure 2. The characteristic band at 3492 cm^−1^ and 1642 cm^−1^ represent the stretching and the bending modes of the –OH group of adsorbed water present on the surface of the nanocomposite, respectively [23,35]. The bands in the range 400–880 cm^−1^ correspond to the vibrations of the Fe–O and Zr-O from Fe_3_O_4_ and ZrO_2_, respectively [35]. Moreover, the absorptions at 1036 cm^−1^ and 776 cm^−1^ were assigned to the Si-O-Si and Si-O vibrational band from the APS ligand, respectively [23,36]. The bands at 2959 cm^−1^ and 1470 cm^−1^ were attributed to the bending and stretching modes of -CH_2_- in APS [36]. The absorption peaks at 1620 cm^−1^ and the shoulder peak at 3456 cm^−1^ was ascribed to the bending mode of N-H present in the APS structure [23,36].

**Figure 3:**
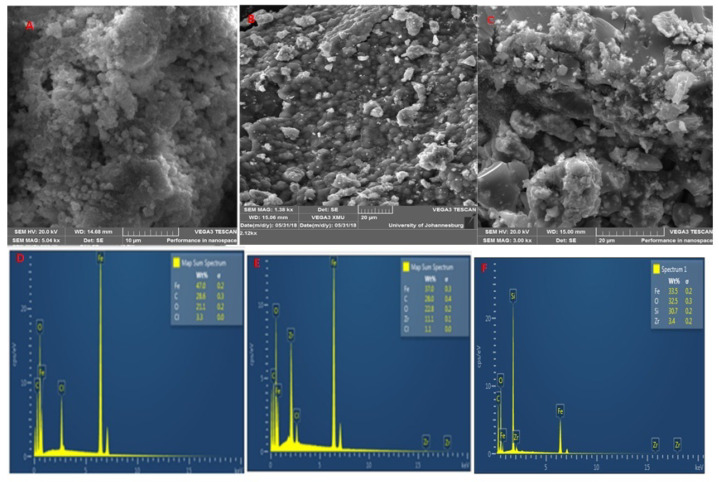
SEM images of (**A**) Fe_3_O_4_ nanoparticles, (**B**) Fe_3_O_4_-ZrO_2_ and (**C**) Fe_3_O_4_-ZrO_2_@APS nanocomposites and EDX spectra of (**D**) Fe_3_O_4_ nanoparticles, (**E**) Fe_3_O_4_-ZrO_2_ and (**F**) Fe_3_O_4_-ZrO_2_@APS nanocomposites.

The SEM images of Fe_3_O_4_ and Fe_3_O_4_-ZrO_2_ (Figure 3) were used to examine the surface morphology of the nanomaterials. The Fe_3_O_4_ nanoparticles displayed rhombohedral shapes, whilst Fe_3_O_4_-ZrO_2_ nanocomposite displayed mostly rhombohedral shapes with some spherical shapes. The Fe_3_O_4_-ZrO_2_@APS composite displayed a combination of rhombohedral, and partially spherical shapes emancipating from the combination of the rhombohedral Fe_3_O_4_ nanoparticles, spherical-like ZrO_2_ and the APS ligand. However, due to the strong magnetic interactions emancipating from the Fe_3_O_4_ nanoparticles, all nanomaterials prepared appear to be agglomerated [37]. The EDS study confirmed the elemental composition within the Fe_3_O_4_ nanoparticles and Fe_3_O_4_-ZrO_2_ nanocomposites. As it can be seen, Fe_3_O_4_-ZrO_2_ nanocomposite showed all the expected Fe, Zr and O elements. These results confirmed that ZrO_2_ was incorporated on the surface of Fe_3_O_4_ nanoparticles. There is however residual chlorine which was possibly left over after washing the synthesized materials with deionized water. Interestingly, the Fe_3_O_4_-ZrO_2_@APS nanocomposite shows no presence of chlorine, which might have been removed on the surface by the powerful ultrasound process prior washing. Furthermore, the presence of the intense Si peak suggests the successful grafting of APS on the Fe_3_O_4_-ZrO_2_ nanocomposite.

**Figure 4:**
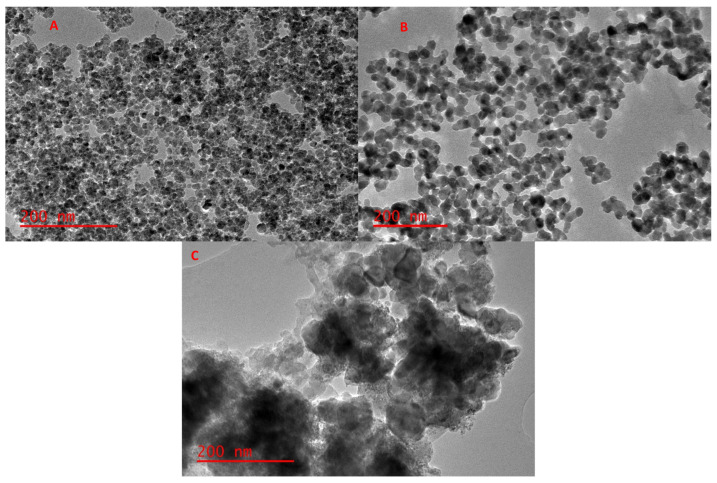
TEM images for the (**A**) Fe_3_O_4_ nanoparticles, (**B**) Fe_3_O_4_-ZrO_2_ and (**C**) Fe_3_O_4_-ZrO_2_@APS nanocomposites.

The TEM images of Fe_3_O_4_ and Fe_3_O_4_-ZrO_2_ are shown in Figure 4. The TEM image for Fe_3_O_4_ displays a uniform structure that is spherical and has agglomerations [38]. The Fe_3_O_4_-ZrO_2_ has a biphasic structure which confirms the successful integration of the Fe_3_O_4_ nanoparticles with ZrO_2_ nanoparticles. These results corroborate the XRD results which suggested that the Fe_3_O_4_ nanoparticles formed aggregates with ZrO_2_ [39]. Similarly, with the SEM results, the Fe_3_O_4_ nanoparticles and Fe_3_O_4_-ZrO_2_ nanocomposite in the TEM images seem aggregated due to their magnetic interactions. Furthermore, the Fe_3_O_4_-ZrO_2_@APS nanocomposite shows a biphasic structure with the incorporated APS ligand. The largely spherical shapes of the ligands can be observed on the TEM image, confirming the grafting of the APS of the Fe_3_O_4_-ZrO_2_ biphasic structure, thus corroborating with the SEM results. As expected, the e_3_O_4_-ZrO_2_@APS nanocomposite was also agglomerated due to the presence of the magnetic Fe_3_O_4_ nanoparticles. The thickness of the APS ligand was estimated as 145 nm on average.

The data for zeta potential performed at different pH value revealed that the increasing of pH increases the negative zeta potential of the composite. It was observed that the point of zero charge (PZC) of the composite was 6.9 (Figure S2). This suggested that above this value, the composite preferred the adsorption of the positively charged trace metal cations. The PZC of 6.9 obtained in this study agreed with the values of 6.4–7.4 reported in the literature [34,38].

### 2.2. Preliminary Studies

Preliminary studies comparing experimental results between the adsorption capacities observed with and without sonication were conducted. These studies were conducted under the same conditions, that is, 7, 100 mg, 10 mg L^−1^ and 30 min pH, mass of adsorbent, initial concentration of each analyte and contact time, respectively. The adsorption process was assisted by ultrasonic system and orbital shaker. The data obtained was expressed as adsorption capacity and it is presented in Table 1. The major difference in the adsorption capacity with and without sonication suggest that ultrasound plays a big role in the adsorption of target metals. This also suggested that the ultrasonic waves increased the interaction between the metals and the surface of the adsorbent, thus increasing the adsorption ability of magnetic sorbents.

### 2.3. Optimization Strategy

**Figure 5:**
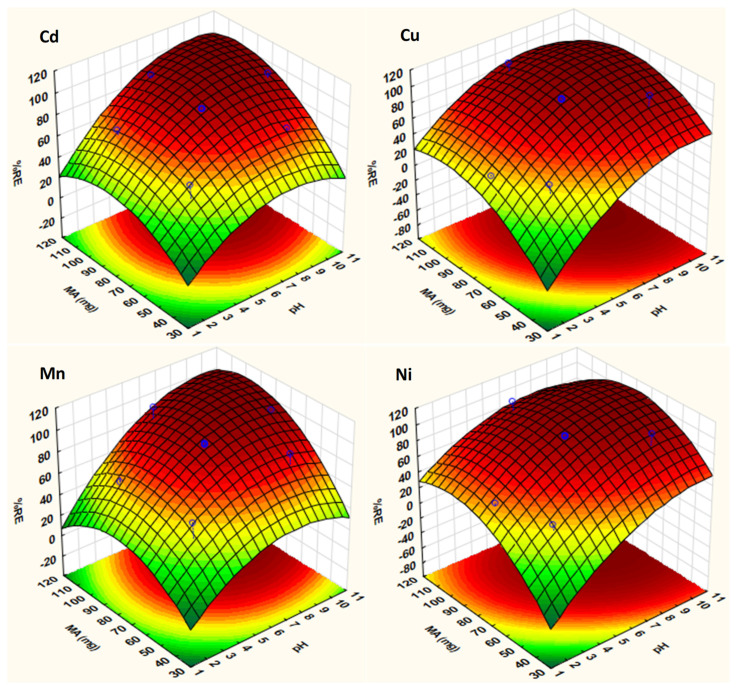
Response surface plots for the CCD obtained for each element.

The three-dimensional (3D) response surface plots were used to explain the effect the pair of variables (sample pH and mass of adsorbent) involved in the adsorption of metals (Figure 5). The curvatures seen in these plots has been ascribed to the interaction between the two variables. As seen in Figure 5, the adsorption of Cd, Cu, Mn and Ni increased as the pH of the solution increases, and the maximum adsorption was achieved when the pH of the solution was above the PZC value. In addition, removal percentage has positive correlation with the mass of the adsorbent such that the adsorption increased with increasing mass of the adsorbent. These finding confirmed the strong participation of the ultrasound power in mass transfer. Therefore, based on the 3D plots and quadratic equations, the optimum conditions were found to be pH 7 and MA 91 mg.

#### Validation of the Optimized Conditions

According to the RSM optimization model, the optimum values for the independent variables are sample pH 7 and mass of adsorbent 91 mg. The maximum removal efficiencies for Cd, Cu, Mn and Ni were 99.8, 100, 99.7 and 101%, respectively. Therefore, confirmatory experimental were performed to validate the predicted results obtained using the RSM model. The experimental removal efficiencies values under the optimum conditions (the experiments were carried out in triplicate) were calculated as 99.2 ± 1.2, 99.3 ± 0.8, 99.6 ± 1.3 and 98.6 ± 1.0% for Cd, Cu, Mn and Ni, respectively. It can be observed that the predicted and experimental values were in close agreement.

### 2.4. Adsorption Isotherms

**Figure 6:**
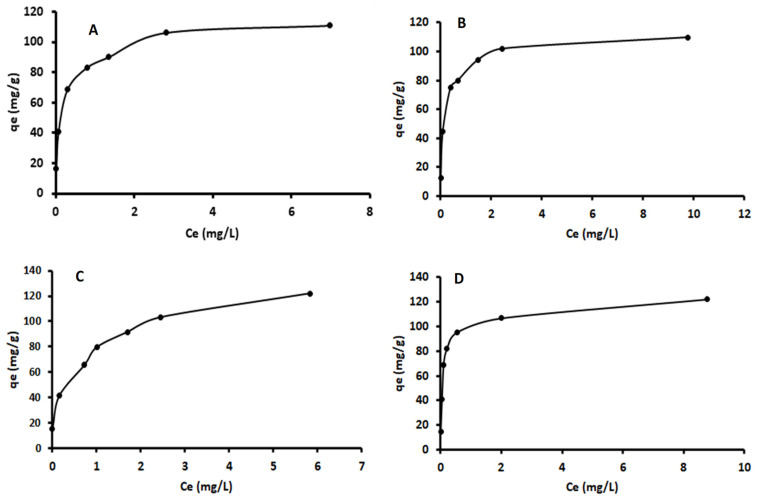
Adsorption of (**A**) Cd, (**B**) Cu, (**C**) Ni and (**D**) Mn onto Fe_3_O_4_-ZrO_2_@APS adsorbent.

The effect of initial concentration of each metal on adsorption by Fe_3_O_4_-ZrO_2_@APS was evaluated under optimal conditions and the data is presented in Figure 6. The results obtained the adsorption capacities increased with increasing concentration and at higher concentrations, the adsorption rate reached a plateau. Using the data in Figure 6, the interaction between the sample solution containing analytes of interest and the adsorbent were described using the adsorption isotherms. In addition, the isotherms were used to determine the maximum adsorption capacity of Fe_3_O_4_-ZrO_2_@APS to adsorb Cd, Cu, Mn and Ni.

The batch adsorption data were analyzed using the Langmuir and Freundlich model (Equations (1)–(3)):

Linearized Langmuir isotherm model:

(1) $$\frac{C_{e}}{q_{e}}=\frac{1}{q_{\max}}C_{e}+\frac{1}{K_{L}q_{\max}}$$

where *K_L_*, is the Langmuir constants (L mg^−1^), *q*_max_ is the maximum amount of the heavy metal adsorbed per unit weight of Fe_3_O_4_-ZrO_2_@APS nanocomposite, *q_e_* is the uptake at equilibrium, *C_e_* is the equilibrium concentration (mg L^−1^) of trace metals and *C*_0_ is the initial concentration (mg L^−1^) of trace metals. The information from the Langmuir parameters were used to investigate the dimensionless constant separation factor *R_L_*:

(2) $$R_{L}=\frac{1}{1+K_{L}C_{0}}$$

Linearized Freundlich isotherm model:

(3) lnqe=lnKF+1nlnCe

where *K_F_* is the Freundlich constant and *n* is the heterogeneity coefficient.

The adsorption isotherm parameters summarized in Table 2 were calculated from the linearized isotherm models (Figures S3–S6). As seen, the coefficients of determination (R^2^) ranged from 0.9961–0.9994, signifying that the data for adsorption studies fitted well to the Langmuir adsorption model. These findings suggested that the sorption sites on the nanoadsorbent were homogeneous with monolayer adsorption coverage [40]. The Langmuir maximum adsorption capacities were investigated using solutions containing mixed metals. It can be seen from Table 1 that the Langmuir q_max_ values for the metals followed the order Ni > Mn > Cd > Cu. The differences in adsorption capacity values of each metal might be due to their different ability to interact with hydroxyl and amine functional groups on the surface of Fe_3_O_4_-ZrO_2_@APS as well as their hydrate ion radius. According to the literature the adsorption affinity of each metal towards the active sites of the adsorbent is inversely proportional related to the hydrate ion radius [41,42]. Based on the literature, the hydrated ionic radii of Ni, Mn, Cd and Cu are 0.404 nm, 0.438 nm, 0.426 nm, and 0.419 nm, respectively [41,42,43]. The adsorption affinity trend of the investigated metals was somehow consistent with the order of the hydrated ionic radius, except for Mn and Cd. These observations suggested that electrostatic interactions describe the adsorption of metal sorption on the adsorbent, especially for Ni (smallest hydrated ionic radius) and Cu. With respect to Mn and Cd, the results suggest that both the ligand and negatively charged surface (PZC = 6.9 (Figure S2) and optimum pH = 7.0) of the adsorbent on the overall analyte-sorbent interactions. The R_L_ values were used investigate whether the nature of the adsorption isotherms. According to the literature, the isotherm is irreversible, favorable, linear or unfavorable if R_L_ = 0, 0 < R_L_ < 1, R_L_ = 1 or R_L_ greater than 1, respectively [40,44]. As seen in Table 3, the R_L_ values are in the range of 0 < R_L_ <1 indicating that that the adsorption of Cd, Cu, Mn and Ni on Fe_3_O_4_-ZrO_2_@APS are favorable.

The maximum adsorption capacities (mg/g) were 114, 111, 128, and 123 for Cd, Cu, Ni and Mn, respectively. The comparison of sample pH and maximum adsorption capacity obtained using Langmuir isotherm model for adsorptive removal of Cd, Cu, Mn and Ni using Fe_3_O_4_-ZrO_2_@APS with the other adsorbents reported in the literature are presented in Table 2. As seen in Table 3, the Fe_3_O_4_-ZrO_2_@APS has a relatively higher maximum adsorption capacity for the four analytes. In addition, the working conditions such sample pH shows that the current adsorbent can be used efficiently in wastewater treatment.

### 2.5. Kinetics Studies

**Figure 7:**
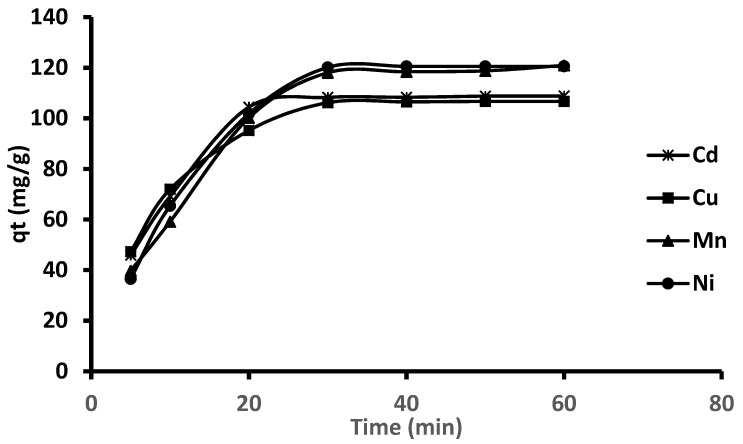
Effect of contact time on the adsorption of Cd, Cu, Mn and Ni by Fe_3_O_4_-ZrO_2_@APS nanoadsorbent.

Figure 7 presents the adsorption capacities of Cd, Cu, Mn and Ni on Fe_3_O_4_-ZrO_2_@APS nanocomposite at different adsorption times. As seen, the adsorption rates were faster at the initial stages and became steadily 30 min. This might be due to the gradual decrease in the active sites of the adsorbent sites and the bulk concentrations of metal ions. The mechanism and the rate determining step of adsorption of Cd, Cu, Ni and Mn ions on Fe_3_O_4_-ZrO_2_@APS nanocomposite were investigated using kinetic (pseudo-first order, pseudo-second order) and intraparticle diffusion models (equations given in Table 4). The rate constants, k and q_e_ values were calculated from the slope of the linear plot of log (q_e_–q_t_) vs. t and t/q_t_ vs. t. The validity of the kinetic models was assessed using correlation coefficient and the closeness of the calculated q_e_ to the experimental q_e_ values. The results in Table 4 showed that the pseudo-second order kinetic model had the highest correlation coefficient (0.9822–0.9955), suggesting that for the removal of mixed metals, the adsorption process is better the described by this kinetic model. In addition, it was observed that the q_e_ values, calculated using pseudo-second-order kinetic model was close to the experimental q_e_ values.

The intraparticle diffusion model was used to investigate the rate controlling step, as well as to understand the influence of mass transfer resistance on the binding of trace metals to the Fe_3_O_4_-ZrO_2_@APS nanoadsorbent [44]. The intraparticle rate constant k_id_ (mg g^−1^ min^1/2^) and intercept C are tabulated in Table 4. The plots showed two linear sections revealing that more than one stage involved in the adsorption of target analytes. It has been reported that if the intraparticle diffusion is the only rate-determining step, then the plot should pass through the origin, otherwise the adsorption process is also affected by the boundary layer diffusion [52]. The results presented in the form of the linear curve showed deviations from the origin. This was because of the differences in the mass transfer rate in the initial step and intraparticle diffusion in final stage of adsorption process [52]. According to literature, the first linear portion is due to a macropore diffusion process and the second portion can be attributed to the micropore diffusion process [53].

The values of the intraparticle model parameters in Table 4 revealed that the external mass transfer was faster than intraparticle diffusion, suggesting that the intraparticle diffusion stage is a slow process [44,54]. Conclusively, since the plots were not passing through origin, this demonstrated that the intra particle diffusion is not the only rate determining factor.

In view of the intraparticle diffusion model results, the Boyd model was used to investigate whether the adsorption process was governed by film diffusion or intraparticle diffusion mechanism [52]. Film diffusion occurs when the adsorbates (metal ions) are transported to the external surface of adsorbent, while intraparticle diffusion happens when adsorbates move within the pores of the adsorbent [53]. The Boyd model expression is given in Table 4. The Boyd plots of B_t_ vs. t resulted to a multilinearity, for this reason only the first linear region (up to 30 min) was used to obtain the slope and intercept [53]. It can be seen in Table 4 that the linear plot did not passes through the origin because the intercept was non-zero. This implies that that the diffusion may be controlled by film diffusion or in combination with other mechanisms [54,55].

### 2.6. Adsorption Mechanism

The PZC of material was found to be 6.9 (Figure S1), this means that at pH lower than 6.9, the adsorbent’s surface is positively charged, while at higher pH (i.e., >6.9) the surface of the adsorbent anionic [56]. The optimum pH used for the adsorption was found to be 7, which is greater than the adsorbent’s PZC, thus suggesting that at optimum pH the adsorbent was anionic. This suggests that there are electrostatic interactions between the negatively charged surface of the adsorbent and positively charged metal ions, resulting in the metal ions being retained by the adsorbent. Additionally, the Langmuir isotherm assumes that adsorption of the metal ions occurred via monolayer adsorption on a homogenous surface [9,57,58]. This meant that one positively charged analyte occupies one available negatively charged active site, when the available active sites are fully occupied a maximum adsorption capacity is predicted using the Langmuir equation. These results agreed with the optimization data which showed pH to be a significant factor on the adsorption of the metal ions onto the adsorbent. Similarly, the adsorption kinetics showed that the adsorption of the metal ions onto the adsorbent followed a pseudo second order type, which suggests that the rate-limiting step was chemisorption influenced surface adsorption where physicochemical interactions between the adsorbent and the adsorbate drove the removal process [59]. In addition, the FTIR results (Figure 2), suggest that hydroxyl and amine groups on APS functionalized adsorbent were dominant adsorption sites. Therefore, the oxygen atoms in the hydroxyl groups and nitrogen atoms in amine moiety coordinated with Ni, Mn, Cd and Cu. Thus, suggesting that the additional mechanism could be driven by electrostatic interactions or proton exchange.

### 2.7. Stability and Reusability

**Figure 8:**
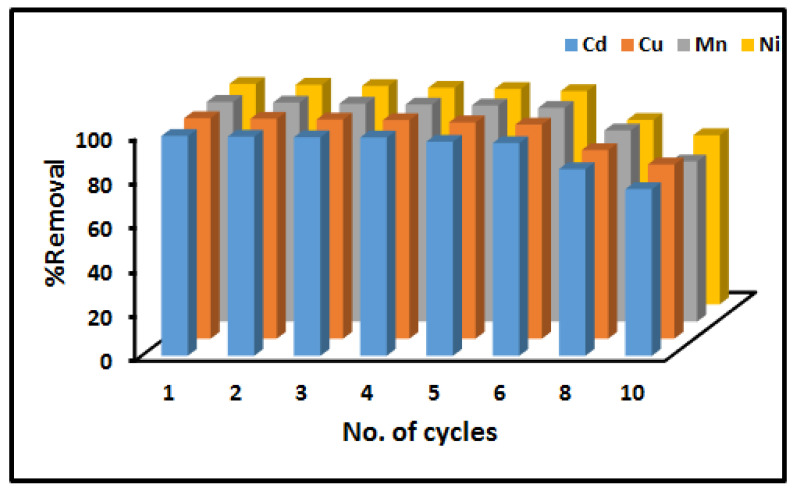
Results obtained from the reusability and regeneration studies.

The stability, regeneration and reusability of adsorbents is one of the important factors as it can reduce the overall cost of the material. Therefore, in this study, the regeneration and reusability of Fe_3_O_4_-ZrO_2_@APS nanocomposite was investigated. The regeneration studies revealed that the nanocomposite can be regenerated by desorbing/washing the metal-loaded adsorbent with 0.5 mol L^−1^ mixture (1:1 ratio) of hydrochloric acid and nitric acid. The percent of removal of metals in aqueous solutions using Fe_3_O_4_-ZrO_2_@APS nanocomposite for ten consecutive cycles is shown in Figure 8. It can be seen from this figure that the percentage removal decreased significantly after the sixth cycle for all the analytes but was still above 70%. These results suggest that the adsorbent has relatively good reusability.

**Figure 9:**
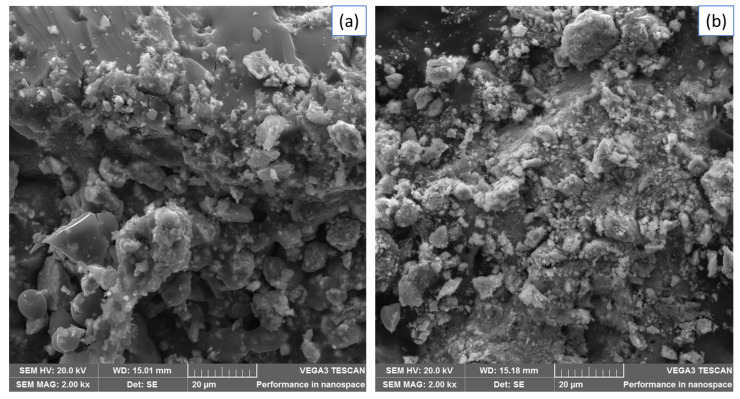
SEM micrographs of Fe_3_O_4_-ZrO_2_@APS (**a**) before and (**b**) after adsorption.

**Figure 10:**
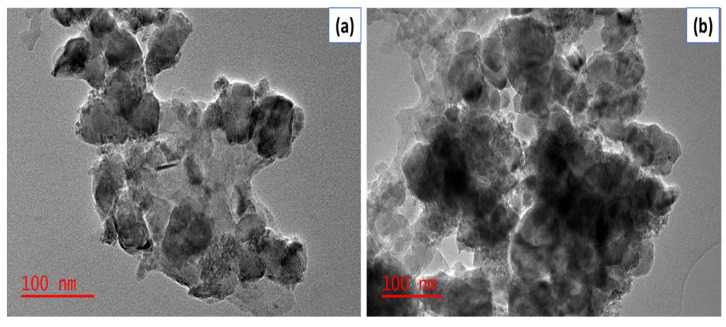
TEM images of Fe_3_O_4_-ZrO_2_@APS (**a**) before and (**b**) after adsorption.

Furthermore, the SEM (Figure 9) and TEM (Figure 10) morphologies of the Fe_3_O_4_-ZrO_2_@APS nanocomposite before and after the adsorption experiments suggest that there was no notable change in the structural properties of the materials.

However, due to the powerful ultrasound process, the agglomerated Fe_3_O_4_-ZrO_2_@APS nanocomposite after regeneration was less agglomerated than the nanocomposite before regeneration. Furthermore, the APS ligand seems to have reduced drastically from 145 nm average thickness to 76 nm. This could be ascribed to the disaggregation of the APS ligand due to the powerful waves generated by the ultrasound., as observed in the TEM images in Figure 10.

### 2.8. Application to Real Samples

The prepared Fe_3_O_4_-ZrO_2_@APS nanocomposite was used as adsorbent for the removal of Cd, Cu, Mn and Ni in acid mine drainage effluent (AMDE1 and AMDE2), wastewater (WW1 and WW2) and river water (RW) samples collected from different areas in Gauteng, South Africa. The concentration of target elements before and after adsorption are presented in Table 5. Some metals were present in trace amounts and were therefore removed completely. The AMD effluent and WW2 samples were found to contain high concentrations of Mn and the adsorbent material performed relative well as the removal efficiency was greater than 90%. These findings revealed that Fe_3_O_4_-ZrO_2_@APS nanocomposite can be used as an adsorbent to remove trace metals from complex matrices such as wastewater and acid mine drainage In addition, it was observed that the performance of the adsorbent was not highly affected by the sample matrix.

## 3. Materials and Methods

### 3.1. Materials and Reagents

All reagents used in this study were of analytical grade unless otherwise stated and double distilled deionized water was used in all the experiments. Absolute ethanol (99.9%), iron(III) chloride hexahydrate (FeCl_3·_6H_2_O) and iron(II) chloride tetrahydrate (FeCl_2·_4H_2_O) salts, zirconium oxalate, ammonium hydroxide solution (28%), ultrapure nitric acid (69%), 3-aminopropyltriethoxysilane and toluene were all purchased from Sigma Aldrich (St. Louis, MO, USA,). Spectrascan single element standard (1000 mg L^−1^) of Mn, Cu, Cd and Ni and the multi-element standard (100 mg L^−1^, Teknolab, VIKEN, Norway) were used to prepare the working standard solutions and the working multi-element solutions for all metal ions, respectively.

### 3.2. Instrumentation

Quantification of Cd, Cu, Mn and Ni in aqueous and real sample was achieved using inductively coupled plasma optical emission spectrometer (ICP-OES, iCAP 6500 Duo, Thermo Scientific, Waltham, Massachusetts, United States,) equipped with charge injection device (CID) detector. A Branson 5800 Ultrasonic Cleaner (Branson, Danbury, CT, USA) was used for the ultrasonic assisted removal of heavy metals. The instrumental detection limits for Cd, Cu, Mn and Ni were 0.1 µg L^−1^, 0.5 µg L^−1^, 0.08 µg L^−1^ and 0.1 µg L^−1^. The scanning electron microscope (SEM, Model Vega 3LMH, TESCAN, Tescan, Brno, Czech Republic) and transmission electron microscope (TEM, JEM-2100F, JEOL Inc, Akishima, Japan) equipped with a LaB6 source were used for assessing the surface morphology of the synthesized adsorbent material. TEM samples were prepared by drop-casting the dispersed material onto a carbon coated copper (Cu) grid. The chemical composition of the functionalized nanocomposite was investigated using the Transform Infrared spectrometer (FT-IR, Spectrum 100, PerkinElmer, Waltham, MA, USA) which was equipped with a Universal Attenuated Total Reflectance (ATR) spectroscopy. X-ray diffraction (XRD, X-ray generator model PW 3710/31, Panalytical (Phillips), Almelo, Netherlands) patterns were characterized for all prepared materials used.

The surface charge of the adsorbent was studied using the Malvern Zetasizer (Nano series, Malvern Instruments, Malvern, UK). The adsorbent was dispersed in deionized water and the pH of the mixture were adjusted to pH ranging from 2–10.

### 3.3. Sampling and Storage

Acid mine drainage effluent samples (AMDE), wastewater influent (WW1) and effluent (WW2) sample were collected in abandoned Princess Gold Mine (Johannesburg, South Africa) and Daspoort wastewater treatment plant (Pretoria, South Africa) The river water samples (RW) were collected from Apies River. All the samples were collected using precleaned plastic containers (500 mL). upon collection, the samples were stored in the fridge at 4 °C until use.

### 3.4. Synthesis of the Adsorbent

#### 3.4.1. Preparation of the Fe_3_O_4_ Nanoparticles

The preparation of Fe_3_O_4_ nanoparticles were prepared according to the method reported by Munonde et al. [23]. A ratio of 2:1 of the salt FeCl_3_∙6H_2_O and FeCl_2_∙4H_2_O was dissolved in deionized water under Argon with vigorous stirring at 85 °C. Then, 50 mL of 25% *v/v* ammonia solution was added quickly to the solution and the color changed immediately from orange to black. The black magnetite was separated using external magnet and then it was washed with deionized water to a final pH of 10. Then finally the magnetite was dried in an oven at 60 °C for 10 h and thereafter it was ground into fine powder using a pestle and mortar.

#### 3.4.2. Preparation of Fe_3_O_4_-ZrO_2_ Nanocomposites

A previously reported method reported by Wu et al. [60] was used with some modification. Briefly; 1.0 g of zirconium oxalate was dissolved in 20 mL of 5:3 (*v*/*v*) mixture of ethanol and water and kept at 80 °C for 2 h with constant stirring. Then 5.0 g of Fe_3_O_4_ nanoparticles were added into the zirconia sol followed by ultrasonication for 1hr. the material was dried for 12 h calcined at 300 °C for 2 h in a muffle furnace.

#### 3.4.3. Functionalization of the Adsorbent

The functionalization of the nanocomposite was carried out according to previous studies [23,36]. The Fe_3_O_4_-ZrO_2_@APS was prepared by first dispersing 1 g of Fe_3_O_4_-ZrO_2_ in 120 mL of toluene on an ultrasonic bath. The dispersion was heated to 60 °C under reflux, with mechanical agitation under nitrogen and 8 mL 3-aminopropyltriethoxysilane was then added. The mixture was refluxed for 3.5 h. The Fe_3_O_4_-ZrO_2_@APS nanocomposite was separated by a magnet then washed with 150 mL of anhydrous ethanol 3 times before it was dried at 60 °C for 3 h.

### 3.5. Experimental Procedure

The simultaneous adsorption of Cd, Cu, Ni and Mn from model sample solutions on to the nanoadsorbent was performed using batch adsorption. The experimental variables were optimized using the central composite design (CCD) matrix and the levels of each factor are presented in Table S1. An aliquot of 20 mL solution containing 10 mg L^−1^ of Cd, Cu, Ni and Mn at an appropriate pH was added in 50 mL sample bottles containing different mass of adsorbent. The mixture was sonicated at room temperature for 60 min. The adsorbent was separated by external margent. The resultant concentrations of the analytes in supernatant were measured using ICP-OES. The percentage removal efficiency (analytical response) of each element was calculated using Equation (4):

(4) $$\%RE=\frac{C_{0}-C_{e}}{C_{0}}\times 100$$

where *C*_0_ and *C*_e_ are initial and equilibrium concentrations of metals.

### 3.6. Optimization Procedure

A response surface methodology (RSM) based on central composite design (CCD) was used to optimize variables that are influential on the adsorption of trace metals. The CCD matrix and analytical responses are presented in Table S2. The results obtained were analyzed using analysis of variance (ANOVA) reproduced in the form of Pareto charts (Figure S2). The analysis of these results (Table S2) by Pareto charts demonstrated that both variables (amount of adsorbent and sample pH) were significant variables with positive effect on the percentages removal of trace metals.

The adsorption isotherm experiments for Cd, Cu, Mn and Ni were conducted simultaneously. Briefly, 250 mL solution containing 5–50 mg L^−1^ of Cd, Cu, Mn and Ni were placed in plastic sample bottles that already contained 91 mg of the adsorbent. The solutions were adjusted to neutral pH 7, and the bottles were sonicated for at 60 min. The adsorbent was separated by external margent and the residual concentration were determined using ICP-OES. The adsorption capacity was calculated using Equation (5):

(5) $$qe=\frac{(C_{0}-C_{e})V}{m}$$

where *q_e_* is the equilibrium adsorption capacity (mg g^−1^), *C_e_* is the equilibrium concentration (mg L^−1^)_,_ *C*_0_ is the initial concentration (mg L^−1^) and *m* is the mass of adsorbent (g)

Kinetic batch experiments were carried out using 50 mg L^−1^ of metal ion stock solution. The experimental conditions were the same as the isotherm batch tests except that the samples were sonicated for 5–60 min. The adsorbent was separated by external margent and the residual concentration were determined using ICP-OES. The adsorption capacity was calculated using Equation (6).

(6) $$qt=\frac{(C_{0}-C_{t})V}{m}$$

where *qt* is the equilibrium adsorption capacity (mg g^−1^) at time *t*, *C_t_* is the equilibrium concentration (mg L^−1^) at time *t*, *C*_0_ is the initial concentration (mg L^−1^) and m is the mass of adsorbent (g).

### 3.7. Regeneration Procedure

The regeneration and reusability of the Fe_3_O_4_-ZrO_2_@APS was done following a previous method by Nyaba et al. [31]. Briefly, the nanoadsorbent already used in the removal procedure was dispersed in 20 mL of a 2.0 mol L^−1^ HCl solution to desorb the adsorbed metals. The mixture was then sonicated for 10 min and washed three times with deionized water. The nanoadsorbent was separated from solution by magnetic decantation and the supernatant was discarded. The Fe_3_O_4_-ZrO_2_@APS adsorbent was dried in oven at 70 °C and then regenerated and reused for up to 10 times.

## 4. Conclusions

The capabilities of Fe_3_O_4_-ZrO_2_@APS for the simultaneous removal of Cd, Cu, Mn and Ni in aqueous solutions and wastewater have been successfully evaluated using an ultrasound-assisted adsorption process. The effect of experimental parameters such as pH, mass of adsorbent on the metal ions percentage removal efficiency were investigated using RSM based on CCD. The results reveled that both variables are influential on the adsorption of Cd, Cu, Mn and Ni. The optimum conditions for metal ion adsorption were selected to be pH 7.0 and adsorbent mass 91 mg. Under the optimum conditions, the maximum adsorption capacities of the nanoadsorbent were found to be 114, 111, 128, and 123 mg g−1 for Cd, Cu, Ni and Mn, respectively. The adsorption data fitted best a pseudo second-order kinetic model for all the analytes and the isotherm data could be fitted to a Langmuir model. The as-synthesized adsorbent was found to be reusable up to six times and was effective in the removal of trace metal ions from water samples.

## Figures and Tables

**Table 1:** Effect of sonication on the adsorption of Cd, Cu, Mn and Ni.

	Adsorption Capacity (mg g^−1^)	
	With Sonication	Without Sonication
Cd	8.97 ± 0.01	0.878 ± 0.005
Cu	9.40 ± 0.05	0.579 ± 0.003
Mn	9.19 ± 0.03	0.777 ± 0.005
Ni	9.87 ± 0.04	1.55 ± 0.02

**Table 2:** Langmuir and Freundlich adsorption isotherm parameters.

	Langmuir	Freundlich
**Cadmium(Cd)**	Qmax = 114	K_F_ = 79.7
	K_L_ = 5.18	n = 3
	R_L_ = 0.004–0.03	
	R^2^ = 0.09984	R^2^ = 0.9336
**Cu**	Q_max_ = 111	K_F_ = 78.3
	K_L_ = 5.63	n = 3
	R_L_ = 0.004–0.04	
	R^2^ = 0.9994	R^2^ = 0.9131
**Ni**	Q_max_ = 128	K_F_ = 75.6
	K_L_ = 2.17	n = 3
	R_L_ = 0.009−0.08	
	R^2^ = 0.9961	R^2^ = 0.9851
**Mn**	Q_max_ = 123	K_F_ = 93.1
	K_L_ = 7.36	n = 3
	R_L_ = 0.002−0.03	
	R^2^ = 0.9993	R^2^ = 0.8091

**Table 3:** Comparison for the removal of Cd, Cu, Mn and Ni by different adsorbents.

Analytes	Adsorbent	Adsorption Capacity (mg/g)	pH	Reference
**Cd and Cu**	Iron-Coated Australian Zeolite	3.7–7.6	6.5	[45]
**Cu and Ni**	Sodium Dodecyl Sulphate Coated Magnetite Nanoparticles	24.3 and 41.2	6	[46]
**Cd**	Mno_2_/Gelatin Composites	89.2	6	[47]
**Mn**	Natural Zeolite (1), Natural Zeolite (1.5)	66, 51.5	6	[48]
**Ni**	Glycine Functionalized Graphene Oxide	38.61	6	[49]
**Cd**	acid modified carbon-based adsorbents	1.22 to 2.02	7	[50]
**Mn**	Polyaniline (PAB) Nanocomposite	50.31	10	[51]
**Cd, Cu, Ni and Mn**	Fe_3_O_4_-ZrO_2_@APS	114, 111, 128 and 123	7	Current Study

**Table 4:** Pseudo-first order kinetic model, psedo-secondorder kinetic, intraparticle difussion and Boyd models for metal adsorption.

	Equations	Parameters	Cd	Cu	Mn	Ni
		*q_e_* exp	111	110	122	122
**Pseudo-first order**	$\ln\left(q_{e}-q_{t}\right)=lnq_{e}-K_{2}t$	*k*_1_ (min^−1^)	0.0629	0.0563	0.0778	0.0820
		*q_e_* (mg g^−1^)	46.4	52.7	101	89.5
		*R* ^2^	0.7854	0.8214	0.9267	0.8314
		*k*_2_ (g mg^−1^ min^−1^)	0.436	0.786	0.650	0.105
**Pseudo-second order**	$\frac{1}{q_{t}}=\frac{1}{K_{2}q^{e}{}_{t}}+\frac{1}{q_{e}}t$	*q_e_* (mg g^−1^)	110	108	121	123
		*R* ^2^	0.9912	0.9955	0.9832	0.9822
		*k_id_*_1_ (mg g^−1^ min^1/2^)	26.2	21.2	25.2	26.0
		*C*_1_ (mg g^−1^)	13.1	18.6	17.3	18.2
**Intraparticle diffusion**	$q_{t}=K_{d}t^{\frac{1}{2}}+C$	*R*_1_^2^	0.9996	0.9867	9894	9898
		*k_id_*_2_ (mg g^−1^ min^1/2^)	0.263	0.286	1.17	0.0190
		*C*_2_ (mg g^−1^)	107	105	118	120
		*R*_2_^2^	0.8690	0.8896	0.8319	0.7631
		Slope	0.134	0.110	0.122	0.150
**Boyd model**	$B_{t}=-0.4977-ln\left(1-F\right)$	Intercept	0.671	0.547	0.923	1.16
		*R* ^2^	0.9768	0.9921	0.9679	0.9455

*q_t_*: amount of adsorbate, adsorbed at time *t*; *k*_1_: rate constant; *q_e_*: sorption capacity *k*_2_: second-order constant; *k_id_*: intraparticle diffusion rate constant; *C*: is the value of intercept which gives information about the boundary layer thickness; *F* is equivalent to *q_t_*/*q_e_*, and *B_t_* is mathematical function of *F*.

**Table 5:** The analysis of real water samples.

Analytes	Parameter	Samples				
		AMDE1	AMDE1	RW	WW1	WW2
**Cd**	Initial concentration (µg/L)	0.61 ± 0.02	ND	ND	0.29 ± 0.04	0.23 ± 0.01
	Concentration after adsorption(µg/L)	ND	ND	ND	ND	ND
	%RE	100			100	100
**Cu**	Initial concentration (mg/L)	4.82 ± 0.06	3.45 ± 0.04	1.73 ± 0.03	2.07 ± 0.03	4.79 ± 0.05
	Concentration after adsorption(mg/L)	ND	ND	ND	ND	ND
	%RE	100	100	100	100	100
**Mn**	Initial concentration (mg/L)	33.2 ± 1.3	22.9 ± 1.2	1.27 ± 0.09	7.28 ± 0.11	15.8 ± 0.9
	Concentration after adsorption (mg/L)	2.17 ± 0.02	1.61 ± 0.03	ND	ND	0.16 ± 0.02
	%RE	93.4 ± 2.1	92.9 ± 1.6	100	100	98.9 ± 1.7
**Ni**	Initial concentration (µg/L)	72.6 ± 0.3	83.4 ± 0.1	8.92 ± 0.05	4.52 ± 0.06	91.3 ± 0.7
	Concentration after adsorption (µg/L)	ND	ND	ND	ND	ND
	%RE	100	100	100	100	100

ND = not detected; AMDE1 = acid mine drainage effluent 1; AMDE2 = acid mine drainage effluent 2; WW1 = wastewater 1; WW2 = wastewater 2; RW = river water.

## Data Availability

The data presented in this study are available on request from the corresponding author.

## Supplementary Materials

The following are available online. Figure S1: Pareto Chart for Cadmium(Cd), Copper(Cu), Manganese(Mn) and Nickel(Ni), Table S1. Experimental range and levels of independent variables, Table S2. The central composite design for the two independent variables.

## Sample Availability

Samples of the compounds are available from the authors.
