# Supplementary material for: Adsorptive Removal of Cd, Cu, Ni and Mn from Environmental Samples Using Fe3O4-Zro2@APS Nanocomposite: Kinetic and Equilibrium Isotherm Studies
Source: Molecules. 2021 May 27;26(11):3209. doi: 10.3390/molecules26113209 (PMC8198006; doi:10.3390/molecules26113209)
Supplement: Supplementary file 1 [file molecules-26-03209-s001.zip › molecules-1194988-supplementary.pdf]

Supplementary data

Ultrasound-assisted adsorptive removal of Cd, Cu, Ni and Mn from environmental samples using Fe_3O_4-ZrO_2@APS nanocomposite: Kinetic and equilibrium isotherm studies

Aphiwe Siyasanga Gugushe[1], Anele Mpupa[1,2], Tshimangadzo S. Munonde[1,2], Luthando Nyaba[1,2], Philiswa N. Nomngongo[1,2,3*]

[1]*Department of Chemical Sciences, University of Johannesburg, Doornfontein Campus, P.O. Box 17011, Johannesburg, 2028, South Africa*

[2]*DSI/NRF SARChI: Nanotechnology for Water, University of Johannesburg, Doornfontein 2028, South Africa*

[3]*DSI/Mintek Nanotechnology Innovation Centre, University of Johannesburg, Doornfontein, 2028, South Africa*

Table S1 Experimental range and levels of independent variables

Variables	Minimum (-)	Central point (0)	Maximum (+)
**Sample pH**	3	6	9
**Mass of Adsorbent (MA)(mg)**	50	75	100

Table S2 The central composite design for the two independent variables

Standard Run	Variables		%removal efficiency			
	pH	MA(mg)	Cd	Cu	Mn	Ni
**1**	3	50	61.7	41.5	39.1	61.7
**2**	3	100	70.1	55.7	57.3	55.9
**3**	9	50	75.9	95.0	99.4	87.0
**4**	9	100	92.9	93.4	94.5	91.8
**5**	1.8	75	38.4	33.1	46.9	41.1
**6**	10	75	88.0	88.0	89.0	89.9
**7**	6	40	50.8	39.2	35.4	42.2
**8**	6	110	94.4	93.3	91.5	93.2
**9 (C)**	6	75	92.0	92.9	95.0	93.3
**10 (C)**	6	75	92.0	92.4	94.1	94.3
**11 (C)**	6	75	92.0	93.4	95.0	92.7
**12 (C)**	6	75	92.0	92.4	94.9	93.6

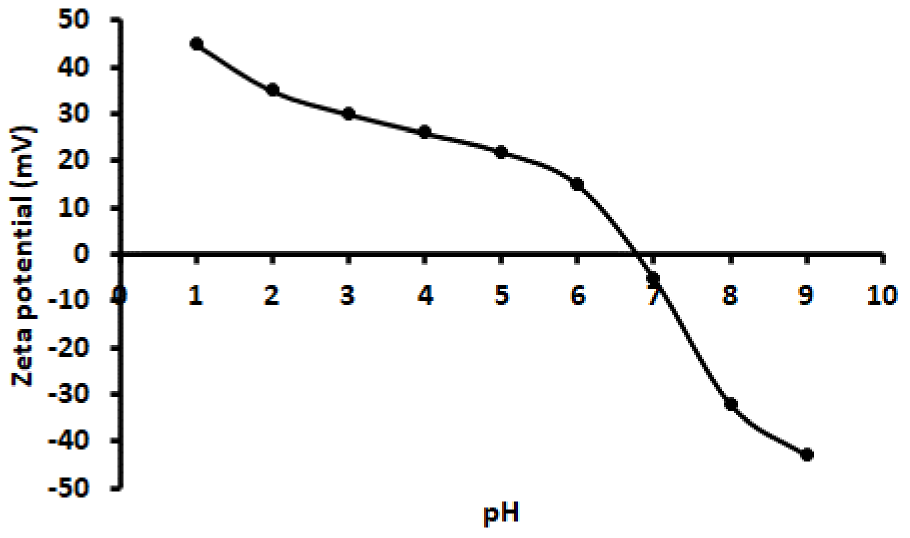

Fig. S1 Determination of point of zero charge

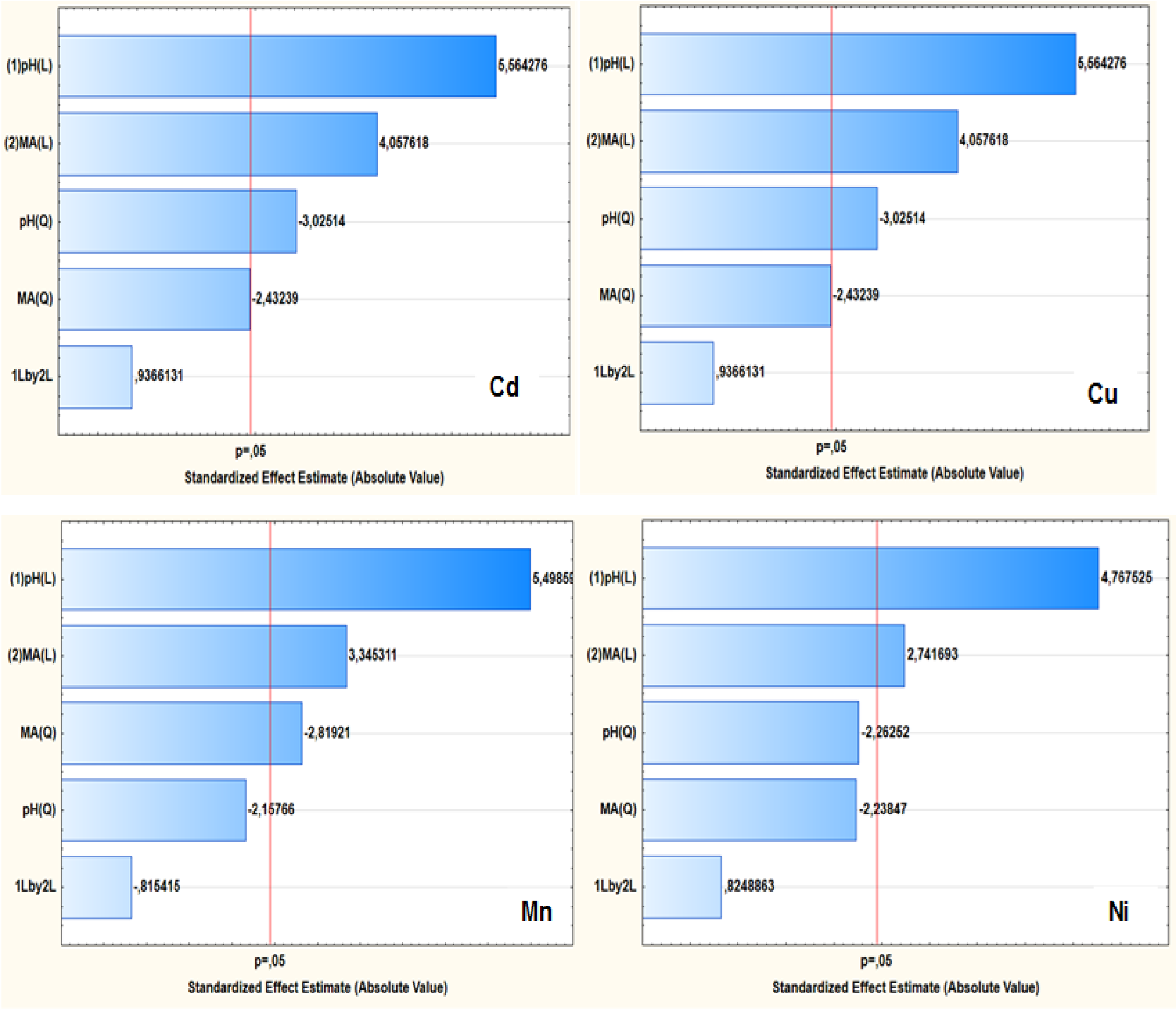

Fig. S2 Pareto Chart for Cadmium(Cd), Copper(Cu), Manganese(Mn) and Nickel(Ni)

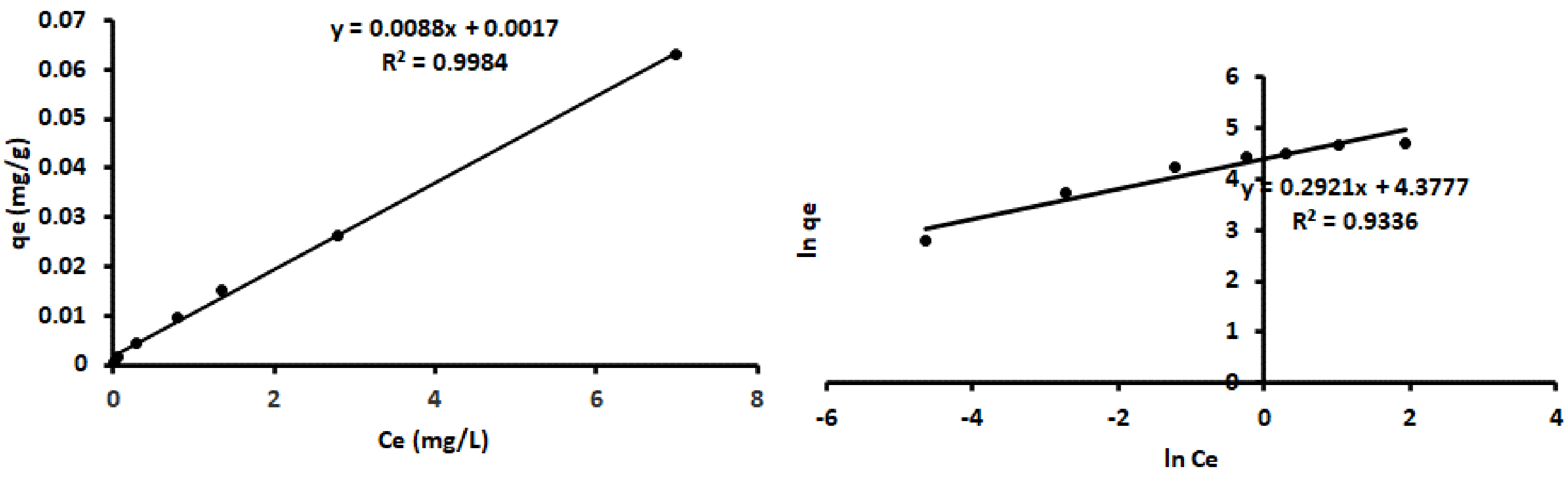

Fig S3 Application of isotherm models to experimental adsorption data of Cd by Fe_3O_4-ZrO_2@APS nanocomposite (a) Langmuir and (b) Freundlich

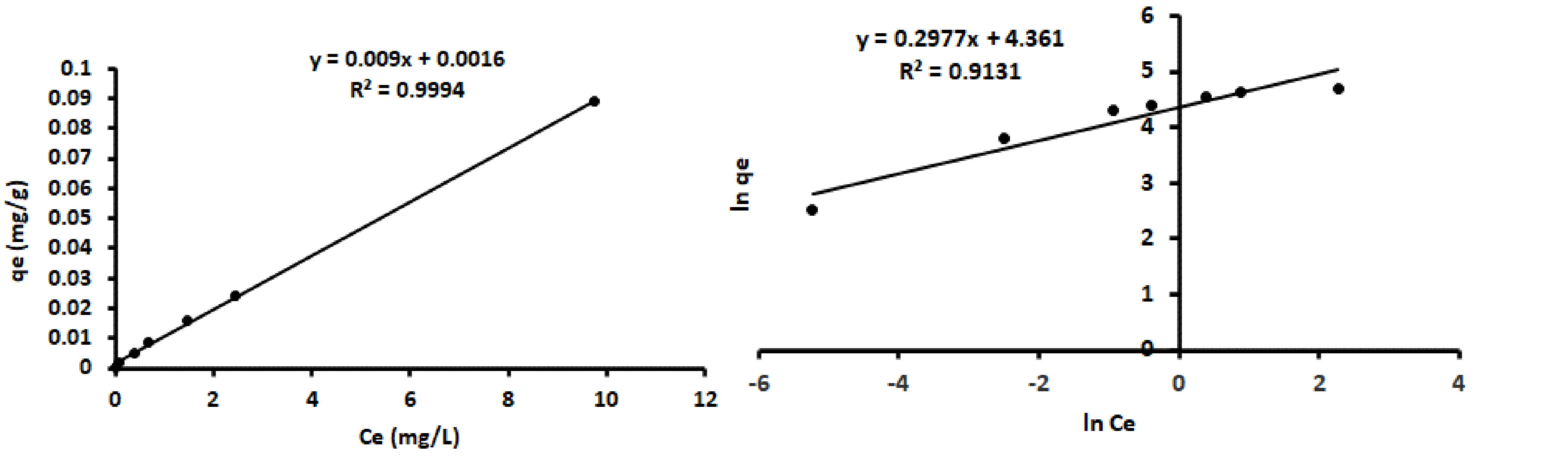

Fig S4 Application of isotherm models to experimental adsorption data of Cu by Fe_3O_4-ZrO_2@APS nanocomposite (a) Langmuir and (b) Freundlich

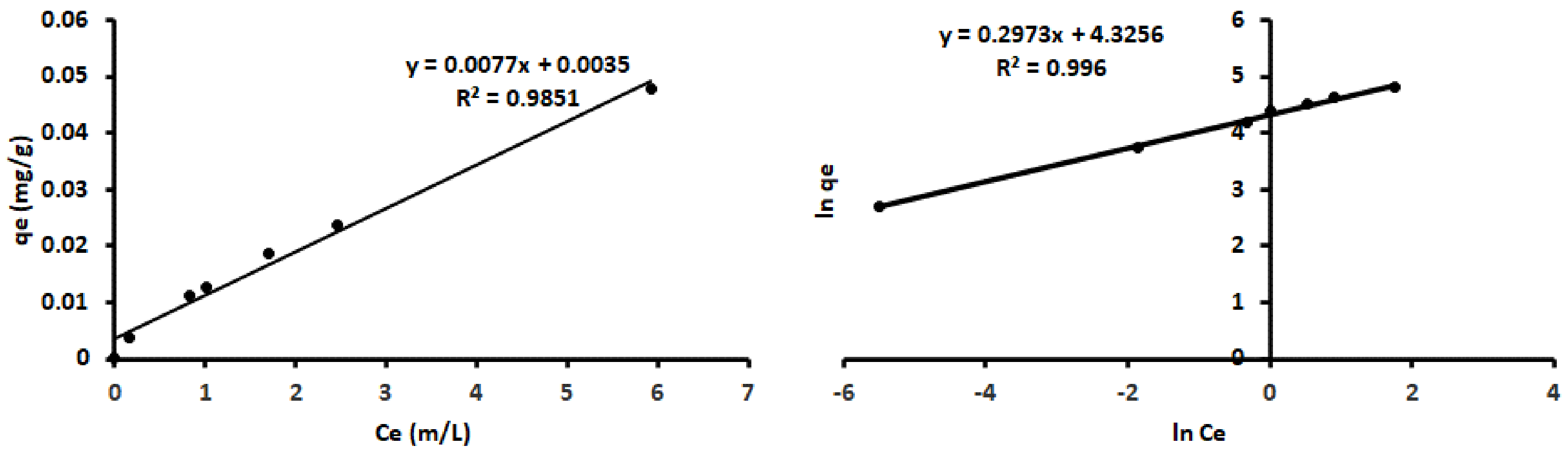

Fig S5 Application of isotherm models to experimental adsorption data of Ni by Fe_3O_4-ZrO_2@APS nanocomposite (a) Langmuir and (b) Freundlich

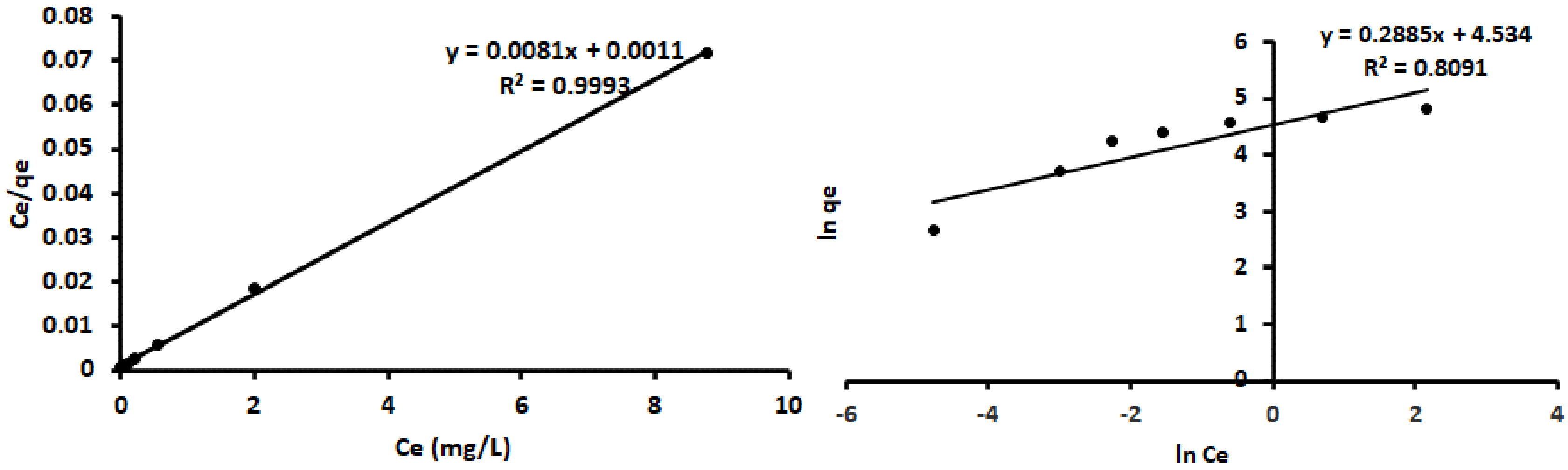

Fig S6 Application of isotherm models to experimental adsorption data of Mn by Fe_3O_4-ZrO_2@APS nanocomposite (a) Langmuir and (b) Freundlich
